# Two New Pentacyclic Triterpene Saponins from the Leaves of *Akebia trifoliata*

**DOI:** 10.3390/molecules21070962

**Published:** 2016-07-22

**Authors:** Qiao-Lin Xu, Jing Wang, Li-Mei Dong, Qiang Zhang, Bi Luo, Yong-Xia Jia, Hong-Feng Wang, Jian-Wen Tan

**Affiliations:** 1 Guangdong Provincial Key Laboratory of Bio-Control for Forest Diseases and Pests, Guangdong Academy of Forestry, Guangzhou 510520, China; qlxu@sinogaf.cn (Q.-L.X.); wanghf@sinogaf.cn (H.-F.W.); 2 Key Laboratory of Plant Resources Conservation and Sustainable Utilization, South China Botanical Garden, Chinese Academy of Sciences, Guangzhou 510650, China; w_j771@163.com (J.W.); donglimei1990@163.com (L.-M.D.); jyx@scib.ac.cn (Y.-X.J.); 3 Guangdong Provincial Key Laboratory of Applied Botany, South China Botanical Garden, Chinese Academy of Sciences, Guangzhou 510650, China; zqiang55@126.com (Q.Z.); bluo023@163.com (B.L.); 4 College of Life Sciences, University of Chinese Academy of Sciences, Beijing 100049, China

**Keywords:** *Akebia trifoliata*, triterpene saponins, α-glucosidase inhibitor, cytotoxicity

## Abstract

Two new pentacyclic triterpene saponins, named akebiaoside K (**1**) and akebiaoside N (**2**), were isolated from the leaves of *Akebia trifoliata*, together with five known triterpenoids **3**–**7**. They were all isolated from the leaves of *A. trifoliata* for the first time. Their structures were established by spectral and chemical means. Triterpenes **5** and **7** were found to show moderate in vitro cytotoxicity against human tumor A549, HeLa and HepG2 cell lines, with IC_50_ values ranging from 0.023 to 0.038 mM. Triterpenes **5**–**7** were further revealed to show significant in vitro α-glucosidase inhibitory activity with IC_50_ values from 0.040 to 0.220 mM, making them more potent than the reference compound acarbose (IC_50_ 0.409 mM). Meanwhile, no obvious inhibitory effects were observed for the isolated triterpene saponins **1**–**4** in both bioactivity assays.

## 1. Introduction

**Figure 1:**
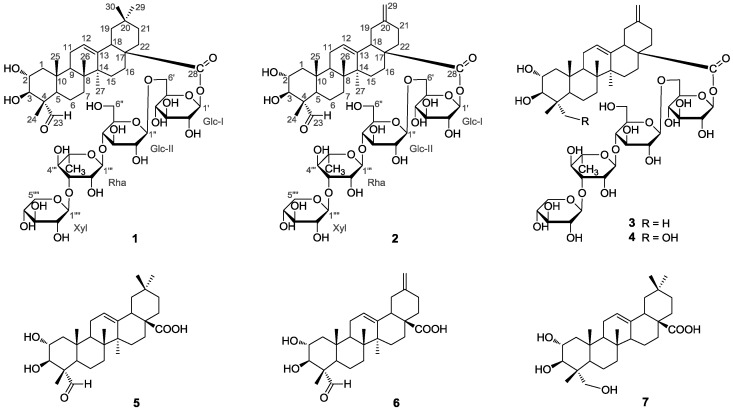
Chemical structures of compounds **1**–**7**.

*Akebia trifoliata* (Thunb.) Koidz. is a perennial, woody liana mainly distributed in the eastern part of Asia [1]. Its fresh fruit, commonly called ‘Ba-Yue-Gua’ in China, has long been consumed by the local people as a delicious food [2]. The air-dried stems and fruits of *A. trifoliata* have traditionally been used in China for centuries as an antiphlogistic, antineoplastic and diuretic agent [3,4]. To date, phytochemical studies have revealed structurally diverse triterpenes, triterpene saponins, phenolics and lignans from this plant, and some of them displayed significant biological activities [5,6,7,8,9,10]. However, those studies were mainly concentrated on the stems and fruits, and seldom focused on the leaves, though our thin layer chromatography (TLC) examination of the ethanol extract showed that it was highly possible that the leaves of *A. trifoliata* could also be a promising source of functional natural products. To examine those potential uncharacterized chemicals in the leaves of *A. trifoliata*, a phytochemical investigation on this plant part was carried out, whereby seven pentacyclic triterpenoids **1**–**7**, including two new triterpene saponins (compounds **1** and **2**) were obtained (Figure 1). Herein, the isolation and structure elucidation of these compounds are described. Besides, compounds **1**–**7** were also tested for their α-glucosidase inhibitory activity and their cytotoxic activity against three human tumor cell lines.

## 2. Results and Discussion

**Figure 2:**
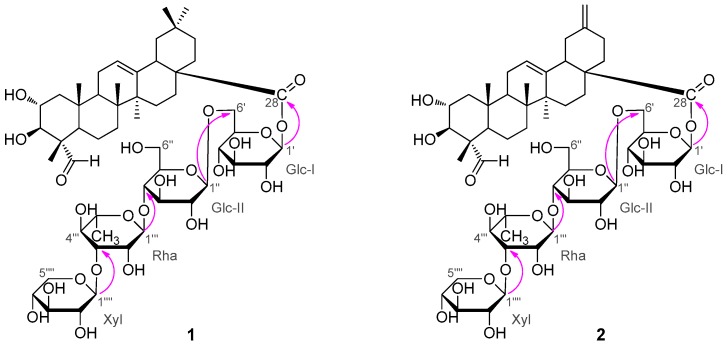
Selected HMBC correlations of compounds **1** and **2**.

Compound **1** was obtained as a white, amorphous powder. Its (−)- and (+)-ESI-MS displayed quasi-molecular ion peaks at *m*/*z* 1087 [M − H]^−^ and 1111 [M + Na]^+^, respectively, corresponding to the molecular formula of C_53_H_84_O_23_, which was confirmed by further HR-ESI-MS (+) analysis ([M + Na]^+^ *m*/*z* 1111.5297, calcd. 1111.5296). The ^1^H- and ^13^C-NMR spectra of **1** exhibited four sugar anomeric protons at δ_H_ 6.22 (Glc-I-1), 4.95 (Glc-II-1), 5.87 (Rha-1), and 5.24 (Xyl-1) (Table 1), and anomeric carbons at δ_C_ 95.5, 104.7, 102.2 and 107.1 (Table 2), suggesting the existence of four sugar moieties in the molecule. Acid hydrolysis of **1** with 2N HCl afforded d-glucose, l-rhamnose and d-xylose in the ratio of 2:1:1, which were identified by GC-MS analysis of their chiral derivatives (see experimental part). The ^1^H- and ^13^C-NMR assignments (Table 1 and Table 2) of the sugar moieties in **1** were established by interpretation of combined HSQC and HMBC data (See the Supplementary Materials). After having excluded the resonances due to the sugar moieties, the remaining signals in the ^1^H-NMR spectrum for the aglycone unit of **1** were readily recognized for six tertiary methyls at δ_H_ 1.42 (3H, s), 1.20 (3H, s), 1.07 (3H, s), 1.04 (3H, s), 0.90 (3H, s) and 0.88 (3H, s), an olefinic proton at δ_H_ 5.40 (1H, t), two oxymethine protons at δ_H_ 4.21 (1H, m) and 4.04 (1H, d, *J* = 10.1 Hz), and an aldehyde proton at δ_H_ 9.61 (1H, s). The ^13^C-NMR spectrum indicated, besides the signals for the sugar moieties, 30 carbons for the aglycone unit, including six methyls, nine methylenes, six methines (including an olefinic methine at δ_C_ 122.4, two oxygenated methines at δ_C_ 67.9 and 76.9 and an aldehyde carbon at δ_C_ 206.3), and nine quaternary carbons (including an olefinic carbon at δ_C_ 144.0 and a carbonyl carbon at δ_C_ 176.3). By comparison, it was found that the ^1^H- and ^13^C-NMR spectroscopic data (Table 1 and Table 2) of the aglycone of **1** were closely related to those of 2α,3β-dihydroxy-23-oxo-olean-12-en-28-oic acid [11], a known triterpene which was also obtained in this study as **5**, with the major difference of the chemical shift value of the carboxyl group at C-28 was shifted from δ_C_ 180.0 in **5** to δ_C_ 176.3 in **1**. These findings supported the fact that **1**, as shown in Figure 1, was a monodesmoside saponin of **5** with an oligosaccharide chain linked at C-28 [12]. This deduction was consistent with the molecular formula of **1**, and further in complete accordance with the 2D NMR spectroscopic data. In the HMBC spectrum, ^1^H-^13^C long-range correlation of H-1′ (δ_H_ 6.22) with C-28 (δ_C_ 176.3) was exhibited, which evidenced the glycoside linkage of Glc-I at C-1′ with the aglycone at C-28. The observation of HMBC correlations of H-1″ (δ_H_ 4.95) with C-6′ (δ_C_ 69.0), of H-1″′ (δ_H_ 5.87) with C-5″ (δ_C_ 77.0), and of H-1″″ (δ_H_ 5.24) with C-3″′ (δ_C_ 83.0) confirmed the sugar sequence as shown in Figure 2. The β-anomeric configuration of the d-glucose (Glc-I and Glc-II) and the xylose (Xyl) moieties were determined on the basis of their coupling constants of ^3^*J*_H1′,H2′_ (8.1 Hz), ^3^*J*_H1″,H2″_ (7.8 Hz) and ^3^*J*_H1″″,H2″″_ (7.5 Hz), respectively [12,13].

The α-configuration of the l-rhamnose (Rha) unit was evidenced by the singlet signal δ_H_ 5.87 (br.s) observed for the anomeric proton H-1″′ [12]. Therefore, **1** was identified as 2α,3β,23,29-tetrahydroxyolean-12-en-28-oic acid-*O*-β-d-xylpyranosyl-(1 → 3)-*O*-α-l-rhamnopyranosyl-(14)-*O*-β-d-glucopyranosyl-(1 → 6)-β-d-glucopyranosyl ester (**1**), trivially named akebiaoside K.

Compound **2** was obtained as a white amorphous powder with molecular formula C_52_H_80_O_23_ as determined by HR-ESI-MS analysis ([M + Na]^+^ *m*/*z* 1095.4965, calcd. 1095.4983). Its spectral features and physicochemical properties suggested **2** was also a triterpenoid saponin. Careful comparison revealed that the ^1^H- and ^13^C-NMR spectral data of the aglycone part of **2** were quite close to those of 2α,3β-dihydroxy-23-oxo-30-norolean-12,20(29)-dien-28-oic acid [14], a known noroleanane triterpene which was also obtained in this study as compound **6**, suggesting that the aglycone unit of **2** was the same as **6**. This assignment was supported by the acid hydrolysis of **2** which furnished the free aglycone identified as **6**, and the monosaccharide compounents concomitantly obtained from the acid hydrolysis of **2** were identified as d-glucose, l-rhamnose and d-xylose in the ratio of 2:1:1 based on GC-MS analysis of their chiral derivatives (see experimental section), supporting the presence of the same four sugar moieties as those in **1**. On the basis of combined analysis of ^1^H-^1^H COSY, HMQC and HMBC spectra, all proton and carbon signals were assigned (Table 1 and Table 2). The β-anomeric configuration of the d-glucose units (Glc-I and Glc-II) and the xylose (Xyl) moiety were determined from their coupling constants of ^3^*J*_H1′,H2′_ (8.2 Hz), ^3^*J*_H1″,H2″_ (7.9 Hz) and ^3^*J*_H1″″,H2″″_ (7.5 Hz), respectively [12]. And the α-anomeric configuration of the l-rhamnose unit was judged by the singlet signal at δ_H_ 5.82 (br.s) for the anomeric proton H-1″′ [12]. In the HMBC spectrum, ^1^H-^13^C long-range correlation of H-1′ (δ_H_ 6.14) with C-28 (δ_C_ 175.6) was displayed, which evidenced the glycoside linkage of Glc-I at C-1′ with the aglycone at C-28. The exhibition of obvious HMBC correlations of H-1′′ (δ_H_ 4.91) with C-6′ (δ_C_ 69.1), of H-1″′ (δ_H_ 5.82) with C-5″ (δ_C_ 76.9), and of H-1″″ (δ_H_ 5.22) with C-3″′ (δ_C_ 83.0) confirmed the oligosaccharide sequence in **2** as shown in Figure 2. Based upon the above information, the strcuture of compound **2** was elucidated as 2α,3β-dihydroxy-23-oxo-29-norolean-12,20(30)-dien-28-oic acid *O*-β-d-xylpyranosyl-(1 → 3)-*O*-α-l-rhamnopy-ranosyl-(1 → 4)-*O*-β-d-glucopyranosyl-(1 → 6)-β-d-glucopyranosyl ester (**2**), trivially named akebiaoside N.

The five known compounds were identified as akemisaponin I (**3**) [7], mutongsaponin B (**4**) [6], 2α,3β-dihydroxy-23-oxo-olean-12-en-28-oic acid (**5**) [11], 2α,3β-dihydroxy-23-oxo-30-norolean-12,20 (29)-dien-28-oic acid (**6**) [14] and arjunolic acid (**7**) [15], by comparison of their spectral data (^1^H- and ^13^C-NMR and MS) to those reported in the literature. They were all isolated from the leaves of *A. trifoliata* for the first time.

Compounds **1**–**7** were evaluated for their in vitro cytotoxicity against human cancer cell lines A549, HeLa and HepG2, using a MTT method as described. The resulting IC_50_ values are displayed in Table 3, compared to adriamycin as positive control. Compounds **5** and **7** showed moderate cytotoxicity against all the three cancer cell lines, with IC_50_ values ranging from 22.7 to 38.1 μM. Meanwhile, no obvious activity was detected for the four saponins **1**–**4**. This result reveals an obvious negative effect on the cytotoxicity when the triterpenes were linked with the linear tetrasaccharide chain at C-28. Comparison of the chemical structures and their cytotoxicity of **5** versus **6** indicated that the replacement of the structure fragment C-20(Me)_2_ by the exocyclic double bond of C-20(29) also had a negative effect on the cytotoxicity.

These compounds were further tested for their α-glucosidase inhibitory activity with acarbose used as a reference compound. Compounds **5**–**7** were revealed to show strong α-glucosidase inhibitory activity, with IC_50_ values of 0.047, 0.220 and 0.040 mM, respectively, which were about two to ten-fold stronger than acarbose (IC_50_ 0.409 mM). These results suggested that triterpenes **5**–**7** from the leaves of *A. trifoliata* are effective α-glucosidase inhibitors with potential value for development as effective hypoglycemic agents for diabetes chemotherapy [16]. Like in the cytotoxicity bioassay, no obvious α-glucosidase inhibitory activity was detected for the four triterpenoid saponins **1**–**4**, indicating that a negative effect of the tetrasaccharide chain linked at C-28 on the α-glucosidase inhibitory activity was also evident.

*A. trifoliata* is a medicinal plant naturally widely distributed in the eastern part of Asia. Its air-dried stems and fruits have long been used in China as an antiphlogistic, antineoplastic and diuretic agent. To date phytochemical investigations have revealed a series of triterpenes and triterpene saponins from this plant species. However, those studies were mainly focused on the stems and fruits, and seldom concentrated on the leaves. Our present study revealed that the leaves of *A. trifoliata* is also rich in triterpenes and triterpene saponins. At the same time, however, it is also demonstrated that much future work is needed to unravel the complexity of the chemical constituents in the leaves of *A. trifoliata*.

## 3. Materials and Methods

### 3.1. General Information

Optical rotation were measured on a Perkin-Elmer 341 polarimeter (Perkin-Elmer, Waltham, MA, USA) with MeOH as solvent at the wavelength of 589 nm and 20 °C to obtain their specific optical rotation [α] values after calculation. ESI-MS data were obtained using a MDS SCIEX API 2000 LC/MS/MS system (Applied Biosystems, Foster City, CA, USA) in both positive and negative ion modes in the range of *m*/*z* 50–1000 after the test solutions were directly injected into the ESI source by a syringe pump. HR-ESI-MS mass spectra were obtained on a Bruker maXis instrument (Bruker Daltonik GmbH, Bremen, Germany) in a positive ion mode after direct injection of the test solutions. Nuclear magnetic resonance (NMR) spectra were recorded on a Bruker Advance 600 instrument (Bruker, Fällanden, Switzerland) or Bruker Ascend-500 spectrometer (Bruker BioSpin GmbH, Rheistetten, Germany). Preparative HPLC was carried out on a CXTH P3000 HPLC pump and a UV 3000 UV-Vis Detector with a Fuji-C18 column (10 μm–100 A, ChuangXinTongHeng Science and Technology Co., Ltd., Beijing, China); medium pressure liquid chromatography (MPLC) was performed using a CXTH P3000 HPLC pump, a UV 3000 UV-Vis Detector and a C18 column (400 × 25 mM i.d., 50 μM, YMC Co. Ltd., Kyoto, Japan).

Column chromatography (CC) was performed with silica gel (200–300 mesh, Qingdao Haiyang Chemical Co., Qingdao, China), YMC ODS-A (50 μm, YMC Co. Ltd.), and Sephadex LH-20 (Pharmacia Fine Chemical Co., Ltd., Uppsala, Sweden). Analytical grade petroleum ether (b.p. 60–90 °C), methanol, ethyl acetate, chloroform, *n*-butanol, acetone were purchased from Tianjin Fuyu Fine Chemical Industry Co. (Tianjin, China); HPLC grade methanol was purchased from J & K Chemical Ltd. (Beijing, China); Fractions were monitored by pre-coated HSGF_254_ TLC (Yantai Jiangyou Silica Gel Co., Ltd., Yantai, China), and spot detection was performed under fluorescent light (λ = 254 nm), and then spraying 10% H_2_SO_4_ in ethanol, followed by heating. Pyridine-*d*_5_, DMSO-*d*_6_, 3-(4,5-dimethylthiazol-2-yl)-2,5-diphenyltetrazolium bromide (MTT) and α-glucosidase were purchased from Sigma Chemical Co. (Sigma-Aldrich, St. Louis, MO, USA). RPMI-1640 medium and fetal calf serum were purchased from Gibco BRL (Gaithersburg, MD, USA). Adriamycin was obtained from Pfizer Italia SRL (Roma, Italy). *p*-Nitrophenyl-α-d-glucopyranoside (PNPG) and acarbose were obtained from Tokyo Chemical Industry Co., Ltd. (Tokyo, Japan).

### 3.2. Plant Materials

The leaves of *Akebia trifoliata* were collected in August 2014, at Sangzhi, Hunan Province, China, were identified by Prof. Fu-Wu Xing at the South China Botanical Garden, the Chinese Academy of Sciences (CAS). A voucher specimen (No. 20140815) was deposited at the Laboratory of Bioorganic Chemistry of the South China Botanical Garden, Chinese Academy of Sciences.

### 3.3. Extraction and Isolation

Powdered air-dried leaves of *A. trifoliate* (dried weight 3.5 kg) were extracted three times with 95% EtOH (10 L × 3) at room temperature for 2 days each time. The extract was concentrated *in vacuo* to give a dark brown residue, which was suspended in 3 L of water and then sequentially extracted with petroleum ether (3 L × 3), ethyl acetate (EtOAc, 3 L × 3) and *n*-butanol (*n*-BuOH, 3 L × 3) to yield a petroleum ether-soluble fraction (31 g), an EtOAc-soluble fraction (168 g), and a *n*-BuOH-soluble fraction (245 g) after condensation to dryness in vacuo. The EtOAc-soluble fraction was subjected to silica gel CC (1000 mm × 105 mm i.d.) eluted with a gradient of CHCl_3_–MeOH (97:3–0:100, *v*/*v*) to give fractions F_1_–F_10_ after pooled according to their TLC profiles. Fraction F_5_ (7.1 g), obtained from the elution of CHCl_3_/MeOH of 85:15 (*v*/*v*), was subjected to a silica gel CC (800 × 50 mm i.d.) and successively eluted with CHCl_3_/MeOH (98:2–90:10, *v/v*) to yield sub-fractions F_5-1_–F_5-6_. Sub-fraction F_5-3_ (1.57 g) was separated by MPLC using MeOH/H_2_O (60:40–100:0, *v*/*v*) system at a flow rate of 10 mL/min, and further purified by passing through a Sephadex LH-20 column (1500 mm × 25 mm i.d.) eluted with MeOH to obtain compounds **5** (5.2 mg) and **6** (3.8 mg). Sub-fraction F_5-5_ (2.3 g) was separated by MPLC using a gradient of MeOH/H_2_O (30:70–80:20, *v*/*v*) at a flow rate of 10 mL/min to obtain six sub fractions (F_5-5-1_–F_5-5-6_), and sub fraction F_5-5-5_ was purified by preparative HPLC with a Fuji-C18 column (10 μm–100 A) using 73% methanol in water (*v*/*v*) as a mobile phase at flow rate of 8 mL/min to give compound **7** (15.6 mg).

The *n*-BuOH-soluble fraction (245 g) was dissolved in water and passed through a HP-20 column eluted with distilled water and 90% EtOH. Column chromatography of the 90% EtOH eluate portion (128 g) on silica gel (200–300 mesh) and elution with a gradient system of CHCl_3_/MeOH (85:15–0:100, *v*/*v*) to give six fractions (G1–G6) after pooled according to their TLC profiles. Fraction G3 (18 g), obtained from the elution of CHCl_3_/MeOH of 85:15 (*v*/*v*), was subjected to a silica gel CC (600 × 50 mm i.d.) and successively eluted with CHCl_3_/MeOH (85:15–40:60, *v*/*v*) to yield five sub-fractions (G_3-1_–F_3-4_). Sub-fraction G_3-2_ (2.7 g) was separated by MPLC using a gradient of MeOH/H_2_O (20:80–100:0, *v*/*v*) at a flow rate of 10 mL/min to obtain nine sub fractions (G_3-2-1_–G_3-2-9_), and G_3-2-2_ was purified by preparative HPLC with a Fuji-C18 column (10 μm–100 A) using a gradient of MeOH/H_2_O (50:50–70:30, *v*/*v*) as a mobile phase at flow rate of 10 mL/min to give compound **2** (6.4 mg). G_3-2-6_ was purified by preparative HPLC with a Fuji-C18 column (10 μm–100 A) using a gradient of methanol in water (45:55–63:37, *v*/*v*) as a mobile phase at flow rate of 10 mL/min to give compound **3** (10 mg). G_3-2-7_ was purified by a Sephadex LH-20 column (1500 mm × 25 mm i.d.) eluted with MeOH to afford compound **1** (12 mg). Fraction G5 was subjected to CC on silica gel (200–300 mesh) eluted with a gradient of CHCl_3_/MeOH (80:20–40:60) and Sephadex LH-20 with MeOH to furnish **4** (4.1 mg).

Compound **1**. White amorphous powder. ${\left[\alpha \right]}_{D}^{20}$: −7.3 (*c* 0.33, MeOH). ESI-MS (+) *m/z*: 1111 [M + Na]^+^; ESI-MS (−) *m*/*z*: 1087 [M − H]^−^. HR-ESI-MS (pos.) *m*/*z*: 1111.5297 (calcd for C_53_H_84_NaO_23_, 1111.5296). For ^1^H-NMR (600 MHz, C_5_D_5_N) and ^13^C-NMR (150 MHz, C_5_D_5_N) data, see Table 1 and Table 2.

Compound **2**. White amorphous powder. ${\left[\alpha \right]}_{D}^{20}$: +5.03 (*c* 0.34, MeOH). HR-ESI-MS (pos.) *m*/*z*: 1095.4965 (calcd for C_52_H_80_NaO_23_, 1095.4983). For ^1^H-NMR (600 MHz, C_5_D_5_N) and ^13^C-NMR (150 MHz, C_5_D_5_N) data, see Table 1 and Table 2.

### 3.4. Acid Hydrolysis of ***1*** and ***2***

Each of compounds of **1** and **2** (3 mg) was heated in 2 M HCl (4 mL) at 90 °C for 2 h. The reaction mixture was extracted with EtOAc (3 × 4 mL). The EtOAc extract was purified by passing through a Sephadex LH-20 column (1500 mm × 25 mm i.d.) eluted with MeOH. By TLC comparison, the free aglycone of **1** was determined to be identical as **5**, while that of **2** was identified as the same as **6**, respectively. The aqueous layer was concentrated under reduced pressure to dryness to give a sugar-containing residue, which was reacted with l-cysteine methyl ester hydrochloride in pyridine at 60 °C for 2 h, then added with *N*,*O*-bis(trimethylsilyl)trifluoroacetamide (BSTFA) and stirred under reflux at 60 °C for 10 h. The supernatant was then analyzed by GC–MS using a GCMS-QP2010 PLUS instrument, HP-5ms capillary column (30 m, 0.25 mm ID), Helium at constant rate of 46.5 cm/s, 1 µL injection volume, injector temperature at 230 °C, temperature program as 2 °C/min to 180 °C, then 20 °C/min to 280 °C. Electron ionization mode was used at 70 eV. In the acid hydrolysate of **1** and **2**, d-glucose, l-rhamnose and d-xylose were confirmed by comparison of their retention times of their derivatives with those of authentic d-glucose (*t*_R_ 11.852 min), l-rhamnose (*t*_R_ 5.292 min) and d-xylose (*t*_R_ 6.842 min) derivatives prepared in the same way, respectively.

### 3.5. Cytotoxic Assay

Compounds **1**–**7** were testeded for their cytotoxity against A549 (human lung adenocarcinoma), HeLa (human cervical carcinoma) and HepG2 (human liver hepatocellular carcinoma) cell lines. The three tumor cell lines were obtained from Kunming Institute of Zoology, Chinese Academy of Sciences. The cytotoxic activity of tested chemicals were assayed according to the MTT method using 96 well plates [17]. Briefly, the cells were cultured in RPMI-1640 medium, supplemented with 10% fetal bovine serum in a humidified atmosphere with 5% CO_2_ at 37 °C. 100 μL adherent cells at the density of 5 × 10^4^ cell/mL was seeded into each well of 96-well cell culture plates and incubated in 5% CO_2_ at 37 °C for 24 h to form a monolayer on the flat bottoms. Then, the supernatant per well was removed and subsequently added with 100 μL fresh medium and 100 μL medium containing a test compound. The plate was then incubated in 5% CO_2_ at 37 °C. After 72 h, 20 μL of 5 mg/mL MTT in DMSO was added into each well and incubated for 4 h. The supernatant per well was carefully removed and 150 μL DMSO was added. The plate was then vortex shaken for 15 min to dissolve blue formazan crystals. The optical density (OD) of each well was measured on a Genois microplate reader (Tecan GENios, Männedorf, Switzerland) at the wavelength of 570 nm. All experiments were performed in triplicate and adriamycin was used as a positive control. In each experiment, each of the tumor cell lines was exposed to the test compound at concentrations 50, 25, 12.5, 6.25, 3.125, 1.5625 µg/mL. The inhibitory rate of cell growth was calculated according to the following formula: Inhibition rate (%) = (OD_control_ − OD_treated_)/OD_control_ × 100%. IC_50_ values were calculated by SPSS 16.0 statistic software. The values were based on three individual experiments and expressed as means ± standard deviation (SD).

### 3.6. α-Glucosidase Inhibition Assay

The *a*-glucosidase inhibitory activity of **1**–**7** were determined spectrophotometrically in a 96-well microtiter plate based on *p*-nitrophenyl-α-d-glucopyranoside (PNPG) as a substrate following the method described in literature with slight modifications [18,19]. In brief, α*-*glucosidase (20 μL, 0.8 U/mL) and various concentrations (500, 250, 125, 62.5, 31.25, 15.625 µg/mL) of tested compounds (120 μL) in 67 mM phosphate buffer (pH 6.8) were mixed at room temperature for 10 min. Reactions were initiated by addition of 5.0 mM PNPG (20 μL). The reaction mixture was incubated for 15 min at 37 °C in a final volume of 160 μL. Then, 0.2 M Na_2_CO_3_ (80 μL) was added to the incubation solution to stop the reaction. The activities were detected in a 96-well plate, and the absorbance was determined at 405 nm (for *p*-nitrophenol). The negative blank was set by adding phosphate buffer instead of the sample via the same way as the test. Acarbose was utilized as positive control. The blank was set by adding phosphate buffer instead of the α*-*glucosidase using the same method. Inhibition rate (%) = [(OD_negative control_ − OD_blank_) − (OD_test_ − OD_test blank_)]/(OD_negative blank_ − OD_blank_) × 100%. IC_50_ values of the samples were calculated using the Microsoft Office Excel soft.

## 4. Conclusions

Seven pentacyclic triterpenoids, including two new triterpene saponins **1** and **2**, were isolated from the leaves of *A. trifoliata*. Their structures were identified by spectroscopic and chemical means, including NMR and HRESIMS. All the compounds were isolated from the leaves of *A. trifoliata* for the first time. Triterpenes **5** and **7** were found to show moderate in vitro cytotoxicity against human tumor A549, HeLa and HepG2 cell lines. Triterpenes **5**–**7** were further revealed to show significant in vitro α-glucosidase inhibitory activity, much more potent than the reference compound acarbose. No obvious inhibitory effects were displayed by the triterpene saponins **1**–**4** in either bioactivity assay. The present study indicate that the leaves of *A. trifoliata* are rich in triterpenoids which are potential functional chemicals worthy of further investigation.

## Figures and Tables

**Table 1:** The ^1^H-NMR (500 MHz) spectral data ((ppm), *J* in Hz) of **1** and **2**.

No.	δ_H_ (1) ^a^	δ_H_ (2) ^b^	No.	δ_H_ (1) ^a^	δ_H_ (2) ^b^
1	2.28 (m), 1.38 (m)	2.25 (m), 1.35 (m)	1′	6.22 d (8.1)	6.14 d (8.2)
2	4.21 (m)	4.20 (m)	2′	4.11 (m)	4.08 (m)
3	4.04 (d, 10.1)	4.00 (d, 9.3)	3′	4.21 (m)	4.18 (m)
5	1.60 (m)	1.56 (m)	4′	4.31 (m)	4.31 (m)
6	1.46 (m), 1.01 (m)	1.43 (m), 0.97 (m)	5′	4.07 (m)	4.15 (m)
7	1.46 (m), 1.21 (m)	1.80 (m), 1.67 (m)	6′	4.65 (10.5), 4.27 (m)	4.61 (m), 4.27 (m)
9	1.86 (m)	1.79 (m)	1″	4.95 (d, 7.8)	4.91 d (7.9)
11	1.99 (m), 1.92 (m)	2.22 (m), 1.99 (m)	2″	3.92 (m)	3.90 (m)
12	5.40 (br.s)	5.39 (t, 3.4)	3″	4.15 (m)	4.11 (m)
15	2.27 (m), 1.10 (m)	2.23 (m)	4″	4.45 (m)	4.39 (m)
16	2.03 (m),1.87 (m)	2.12 (m), 1.92 (m)	5″	3.56 (m)	3.54 (m)
18	3.17 (dd, 13.5, 3.5)	3.06 (dd, 13.3, 4.8)	6″	4.17 (m), 4.05 (m)	4.16 (m), 4.03 (m)
19	1.71 (m), 1.22 (m)	2.54 (m), 2.17 (m)	1′″	5.87 (br.s)	5.82 (br.s)
21	1.32 (m), 1.09 (m)	2.16 (m), 2.05 (m)	2′″	4.85 (br.s)	4.83 (br.s)
22	1.83 (m), 1.73 (m)	1.98 (m), 1.67(m)	3′″	4.61 (dd, 9.4, 2.9)	4.59 (m)
23	9.61 (s)	9.59 (s)	4′″	4.50 (m)	4.47 (m)
24	1.42 (s)	1.39 (s)	5′″	5.09 (m)	5.05 (m)
25	1.04 (s)	0.99 (s)	6′″	1.66 (d, 6.1)	1.63 (d, 6.1)
26	1.07 (s)	1.01 (s)	1″″	5.24 (d, 7.5)	5.22 (d, 7.5)
27	1.20 (s)	1.15 (s)	2″″	4.06 (m)	4.03 (m)
29	0.90 (s)	4.73 (s), 4.66 (s)	3″″	4.12 (m)	4.12 (m)
30	0.88 (s)		4″″	4.17 (m)	4.14 (m)
			5″″	4.24 (m), 3.58 (m)	4.20 (m), 3.55 (m)

^a^ Recorded in C_5_D_5_N at 500 MHz; ^b^ Recorded in C_5_D_5_N at 600 MHz.

**Table 2:** The ^13^C-NMR (150 MHz) spectral data (δ (ppm)) of compounds **1** and **2**.

No.	δ_H_ (1) ^a^	δ_H_ (2) ^b^	No.	δ_H_ (1) ^a^	δ_H_ (2) ^b^	No.	δ_H_ (1) ^a^	δ_H_ (2) ^b^
1	47.4 CH_2_	47.4 CH_2_	22	32.2 CH_2_	37.4 CH_2_	5″	77.0 CH	76.9 CH
2	67.9 CH	67.9 CH	23	206.3 CH	206.3 CH	6″	60.9 CH_2_	61.0 CH_2_
3	76.9 CH	76.9 CH	24	10.5 CH_3_	10.5 CH_3_	Rha		
4	56.4 C	56.4 C	25	17.0 CH_3_	17.0 CH_3_	1′″	102.2 CH	102.1 CH
5	47.9 CH	47.9 CH	26	17.3 CH_3_	17.3 CH_3_	2′″	71.9 CH	71.9 CH
6	20.5 CH_2_	20.5 CH_2_	27	25.9 CH_3_	25.8 CH_3_	3′″	83.0 CH	83.0 CH
7	32.2 CH_2_	32.2 CH_2_	28	176.3 C	175.6 C	4′″	72.7 CH	72.7 CH
8	39.8 C	39.8 C	29	33.0 CH_3_	107.3 CH_2_	5′″	69.8 CH	69.8 CH
9	47.8 CH	47.8 CH	30	23.5 CH_3_		6′″	18.2 CH_3_	18.2 CH_3_
10	38.2 C	38.1 C	Glc-І			Xyl		
11	23.1 CH_2_	23.6 CH_2_	1′	95.5 CH	95.5 CH	1″″	107.1 CH	107.1 CH
12	122.4 CH	122.8 CH	2′	73.6 CH	73.6 CH	2″″	75.5 CH	75.5 CH
13	144.0 C	143.3 C	3′	78.5 CH	78.4 CH	3″″	78.2 CH	78.1 CH
14	42.0 C	42.0 C	4′	70.8 CH	70.6 CH	4″″	70.8 CH	70.8 CH
15	28.0 CH_2_	28.0 CH_2_	5′	77.8 CH	77.7 CH	5″″	67.1 CH_2_	67.1 CH_2_
16	23.7 CH_2_	23.3 CH_2_	6′	69.0 CH_2_	69.1 CH2			
17	46.8 C	47.1 C	Glc-ІІ					
18	41.5 CH	47.3 CH	1″	104.7 CH	104.7 CH			
19	46.0 CH_2_	41.4 CH_2_	2″	75.2 CH	75.2 CH			
20	30.6 C	148.1 C	3″	76.2 CH	76.2 CH			
21	33.8 CH_2_	29.9 CH_2_	4″	77.1 CH	77.1 CH			

^a^ Recorded in C_5_D_5_N at 125 MHz; ^b^ Recorded in C_5_D_5_N at 150 MHz.

**Table 3:** Cytotoxicity of compounds **1**–**7** (IC_50_, µM).

Compounds	A549	HeLa	HepG2
**1**–**4**	>100	>100	>100
**5**	30.19 ± 4.55	22.74 ± 1.96	37.97 ± 7.57
**6**	>100	>100	>100
**7**	33.58 ± 3.13	24.35 ± 1.49	38.14 ± 1.63
Adriamycin	0.69 ± 0.07	0.47 ± 0.06	1.22 ± 0.02

Values represent mean ± SD (*n* = 3) based on three individual experiments.

## Supplementary Materials

The following are available online as supporting information at: http://www.mdpi.com/1420-3049/21/7/962/s1, HR-ESI-MS and NMR spectra data of compounds **1** and **2**.
