# Supplementary material for: Two New Pentacyclic Triterpene Saponins from the Leaves of Akebia trifoliata
Source: Molecules. 2016 Jul 22;21(7):962. doi: 10.3390/molecules21070962 (PMC6274375; doi:10.3390/molecules21070962)
Supplement: Supplementary file 1 [file molecules-21-00962-s001.pdf]

Supplementary Materials: Two New Pentacyclic Triterpene Saponins from the Leaves of *Akebia trifoliata*

Qiao-Lin Xu, Jing Wang, Li-Mei Dong, Qiang Zhang, Bi Luo, Yong-Xia Jia, Hong-Feng Wang and Jian-Wen Tan

In this 'Supporting Information' file for the manuscript "**Two New Pentacyclic Triterpene Saponins from the Leaves of *Akebia trifoliata***", HR-ESI-MS (+), ^{1}H- and ^{13}C-NMR, HSQC, HMBC and NOESY spectra of new compounds **1** and **2** are available here as below.

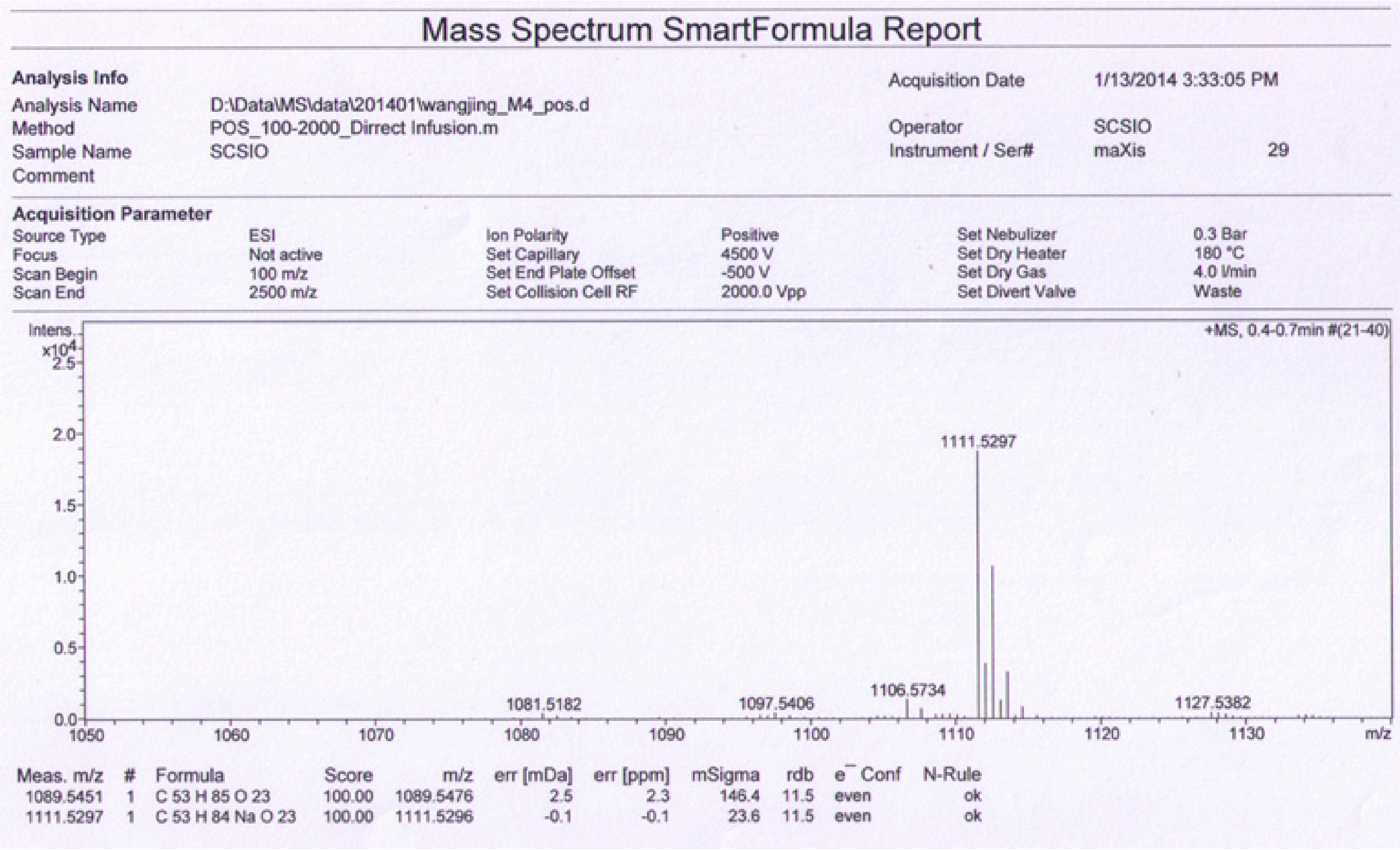

Figure S1. HR-ESI-MS (+) of compound **1**.

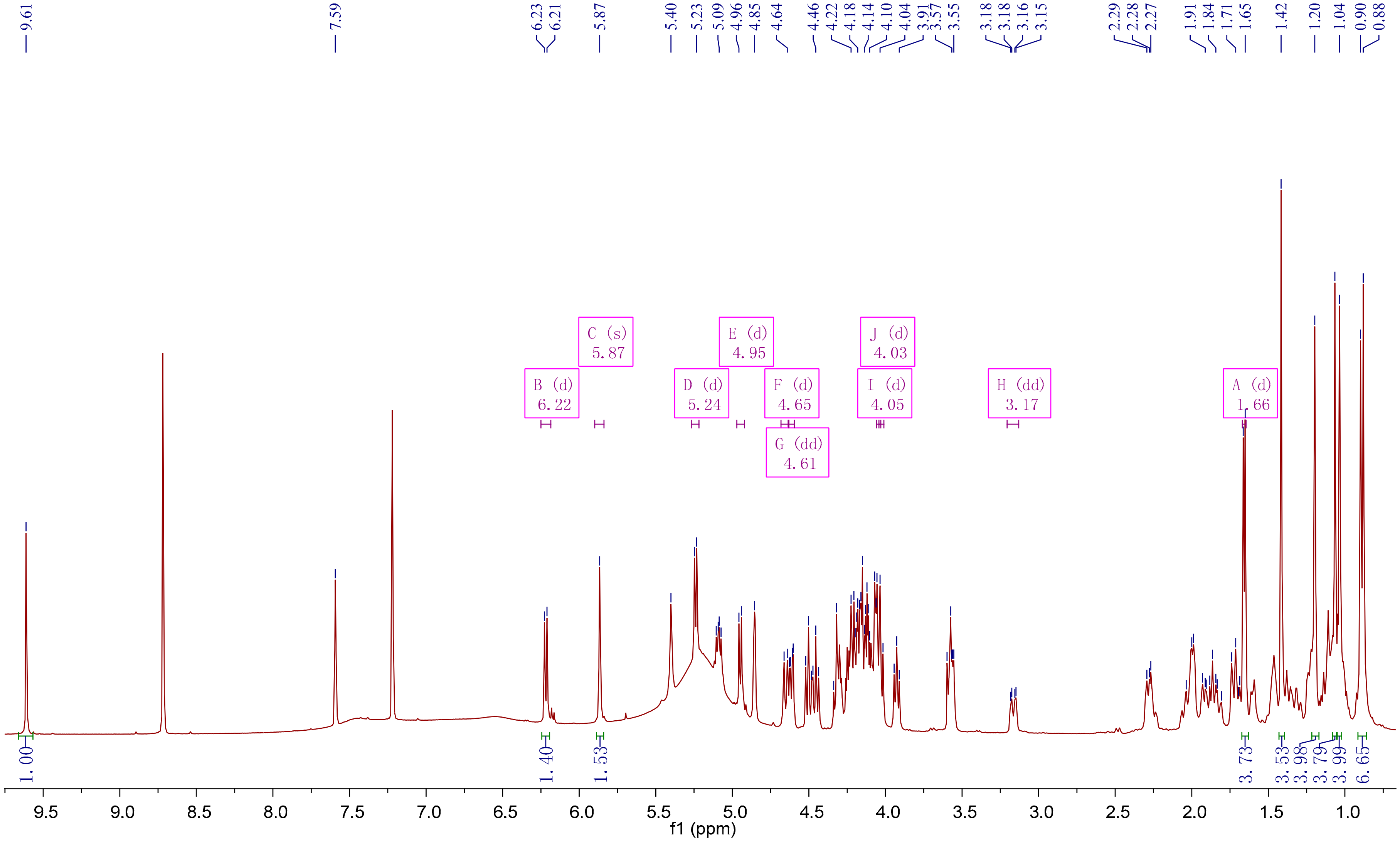

Figure S2. ^{1}H-NMR spectrum of compound **1**.

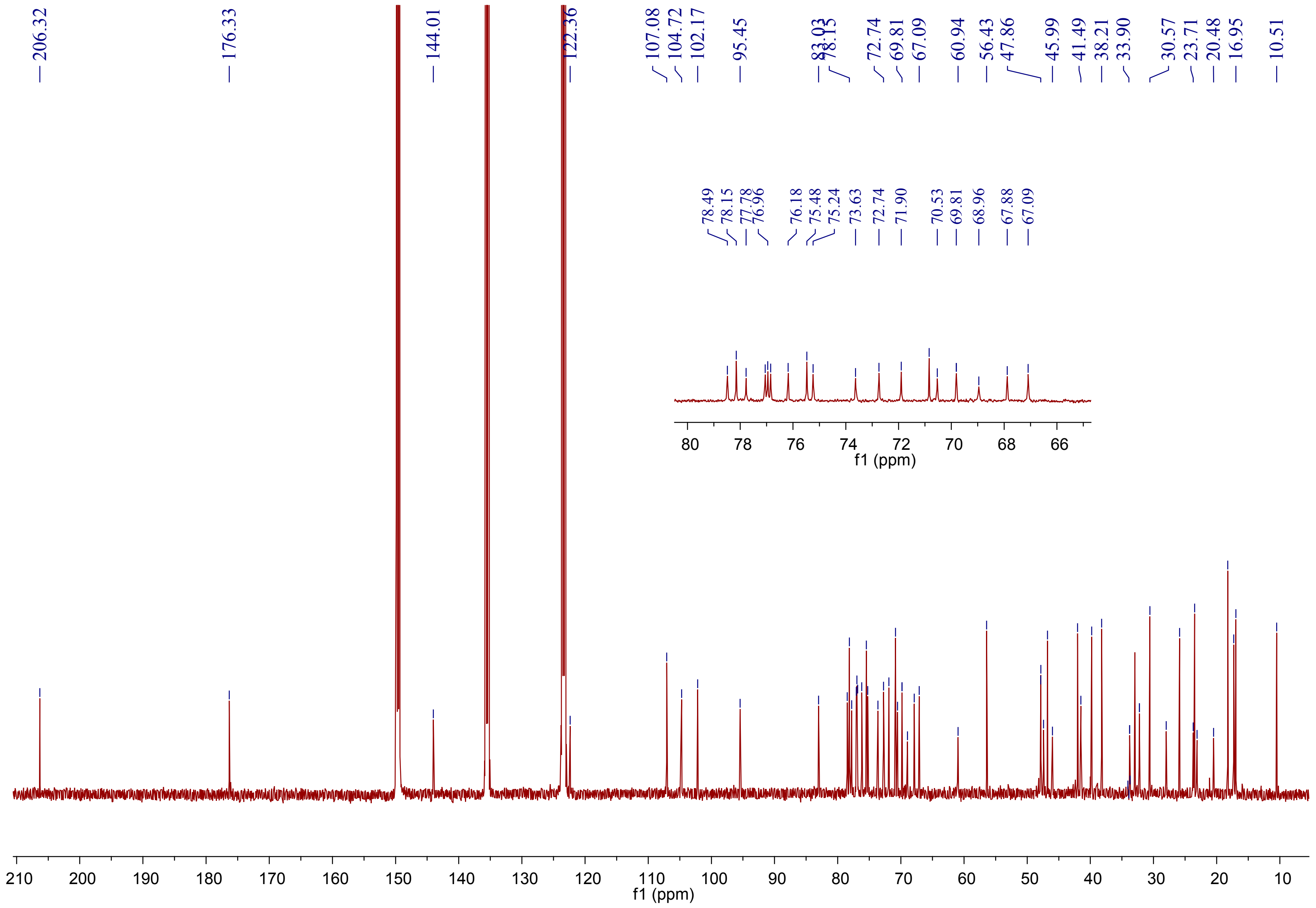

Figure S3. ^{13}C-NMR spectrum of compound **1**.

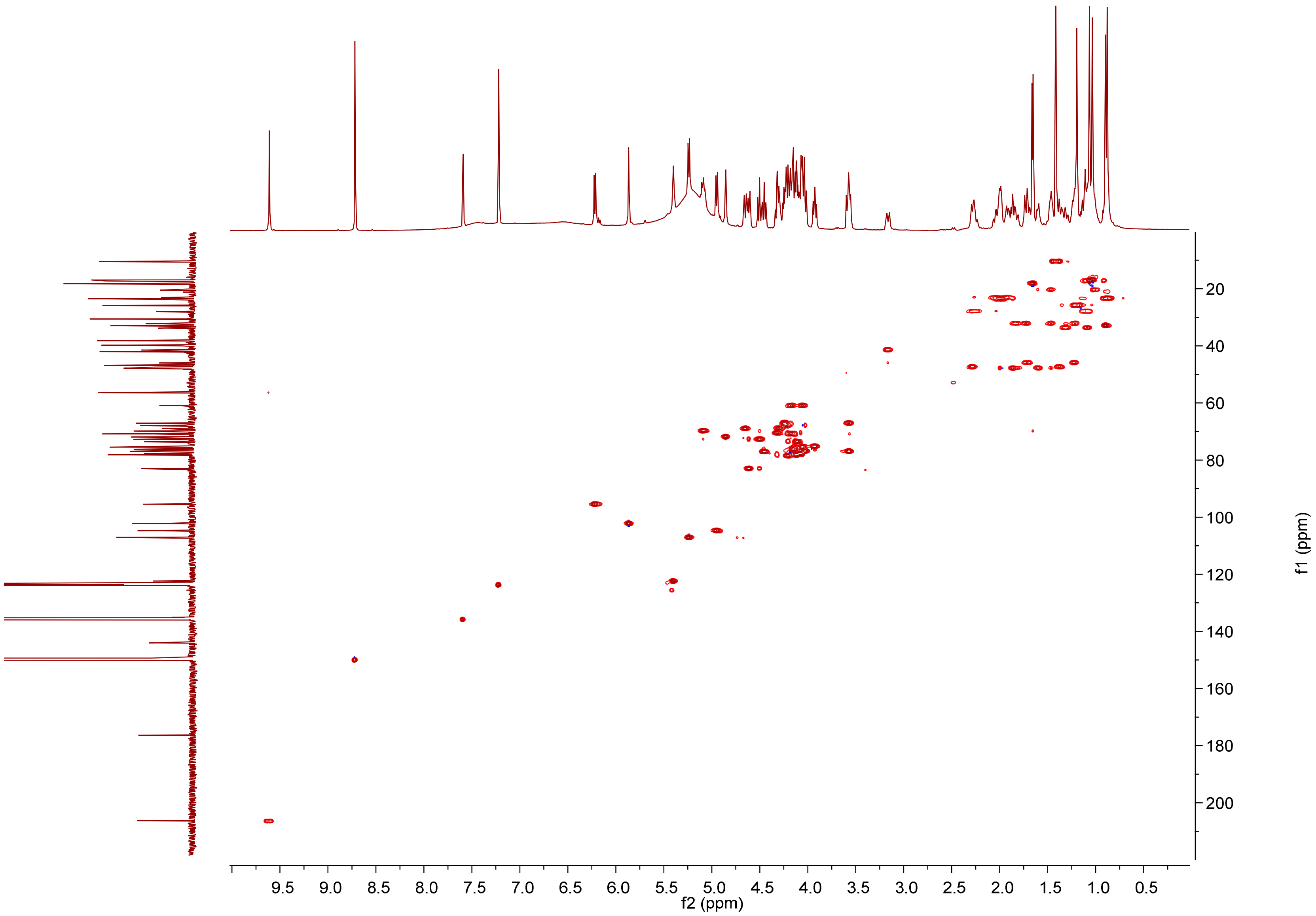

Figure S4. HSQC spectrum of compound **1**.

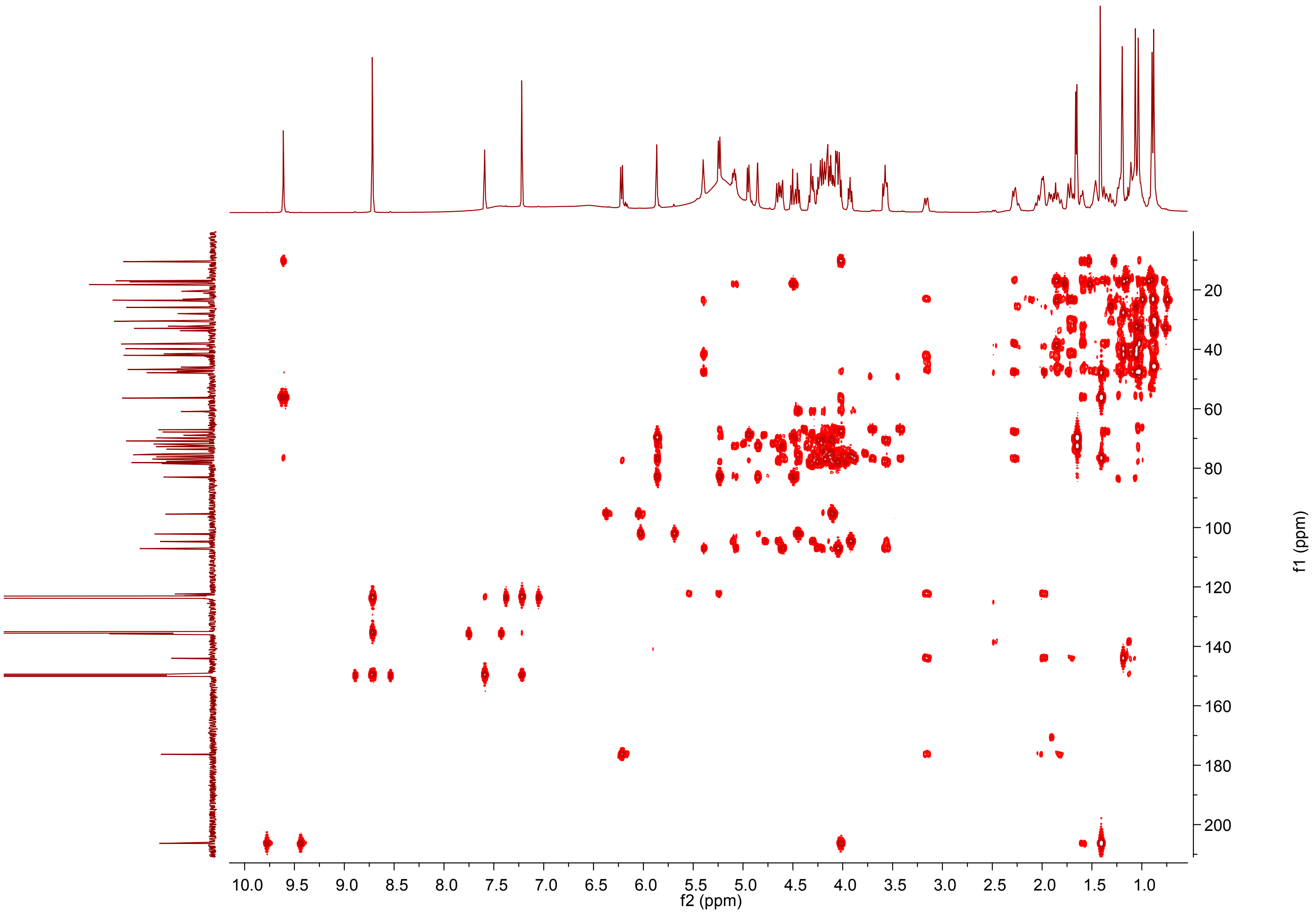

Figure S5. HMBC spectrum of compound **1**.

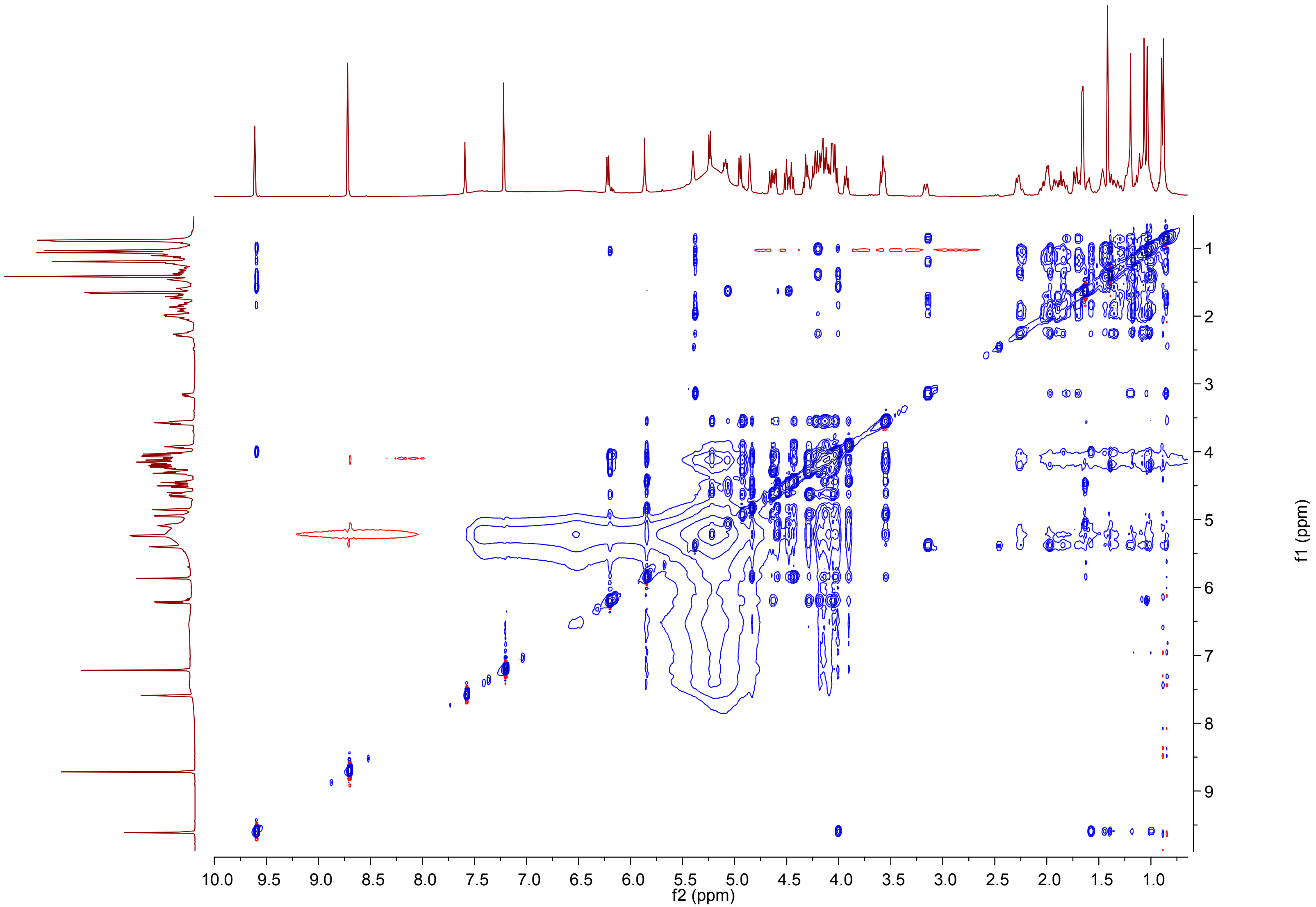

Figure S6. NOESY spectrum of compound **1**.

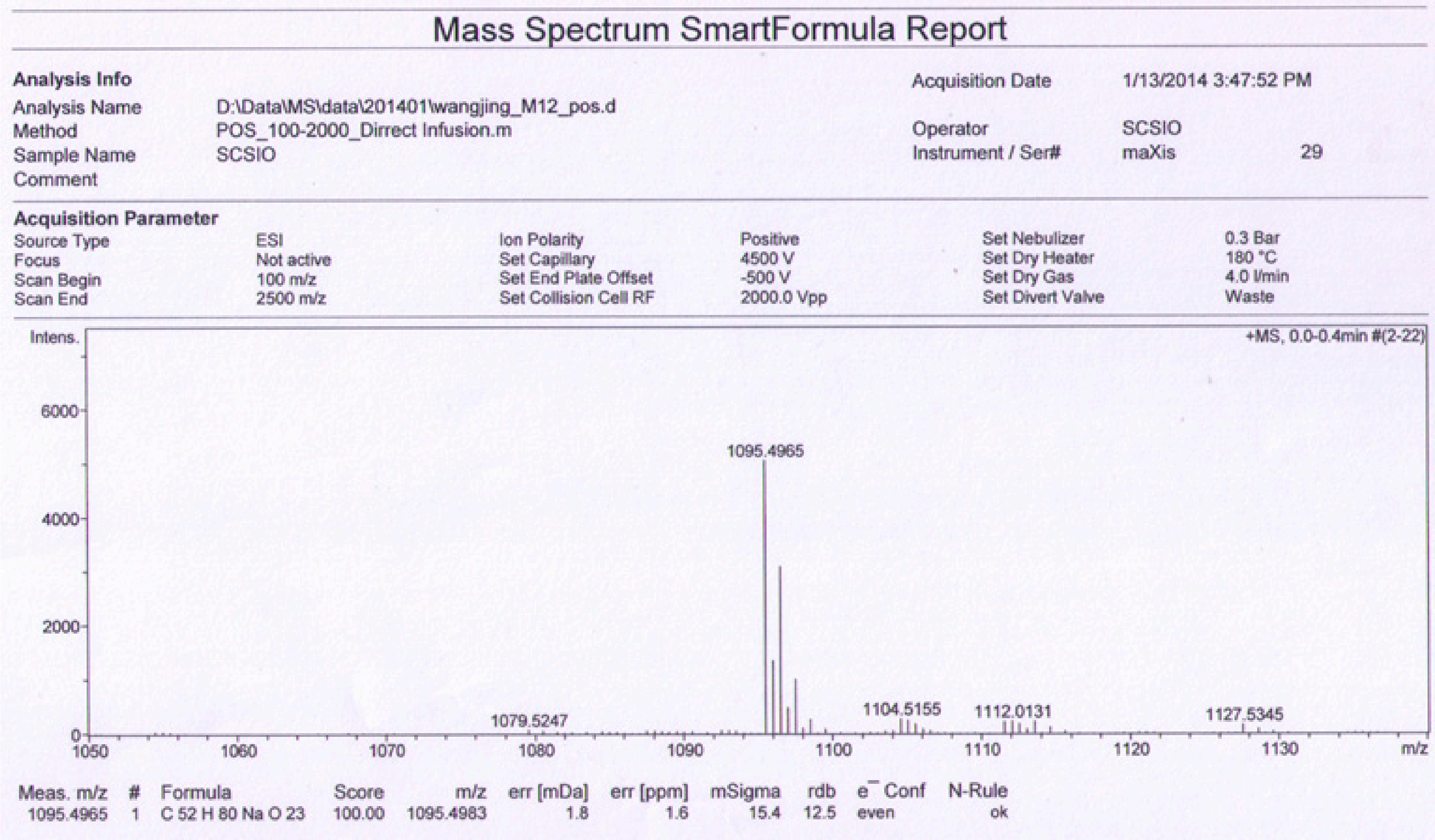

Mass Spectrum SmartFormula Report

Analysis Info

Analysis Name	D:\Data\MS\data\201401\wangjing_M12_pos.d	Acquisition Date	1/13/2014 3:47:52 PM
Method	POS_100-2000_Dirrect Infusion.m	Operator	SCSIO
Sample Name	SCSIO	Instrument / Ser#	maXis 29
Comment			

Acquisition Parameter

Source Type	ESI	Ion Polarity	Positive	Set Nebulizer	0.3 Bar
Focus	Not active	Set Capillary	4500 V	Set Dry Heater	180 °C
Scan Begin	100 m/z	Set End Plate Offset	-500 V	Set Dry Gas	4.0 l/min
Scan End	2500 m/z	Set Collision Cell RF	2000.0 Vpp	Set Divert Valve	Waste

Meas. m/z	#	Formula	Score	m/z	err [mDa]	err [ppm]	mSigma	rdb	e⁻ Conf	N-Rule
1095.4965	1	C 52 H 80 Na O 23	100.00	1095.4983	1.8	1.6	15.4	12.5	even	ok

Figure S7. HR-ESI-MS (+) of compound **2**.

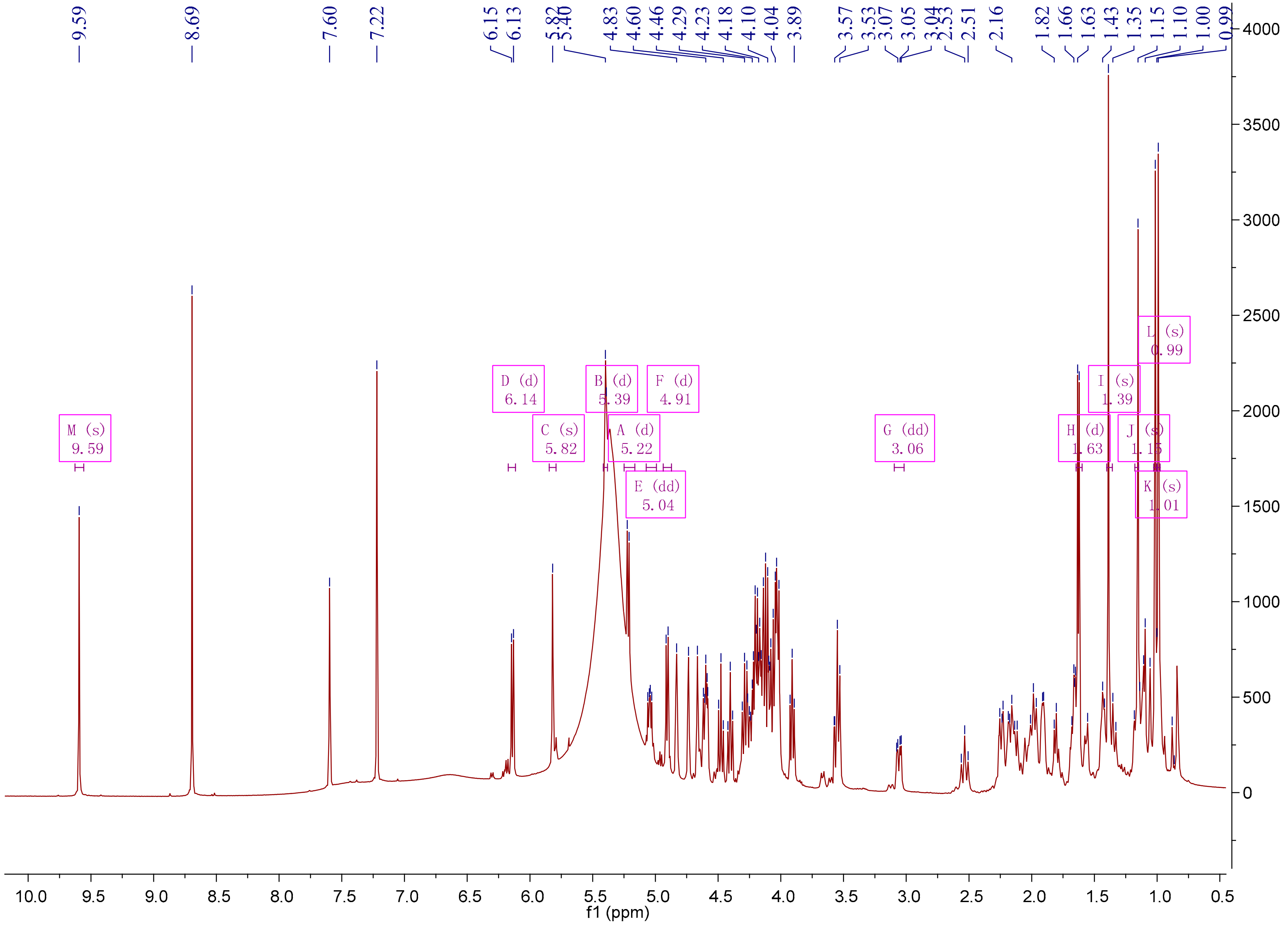

Figure S8. ^{1}H-NMR spectrum of compound **2**.

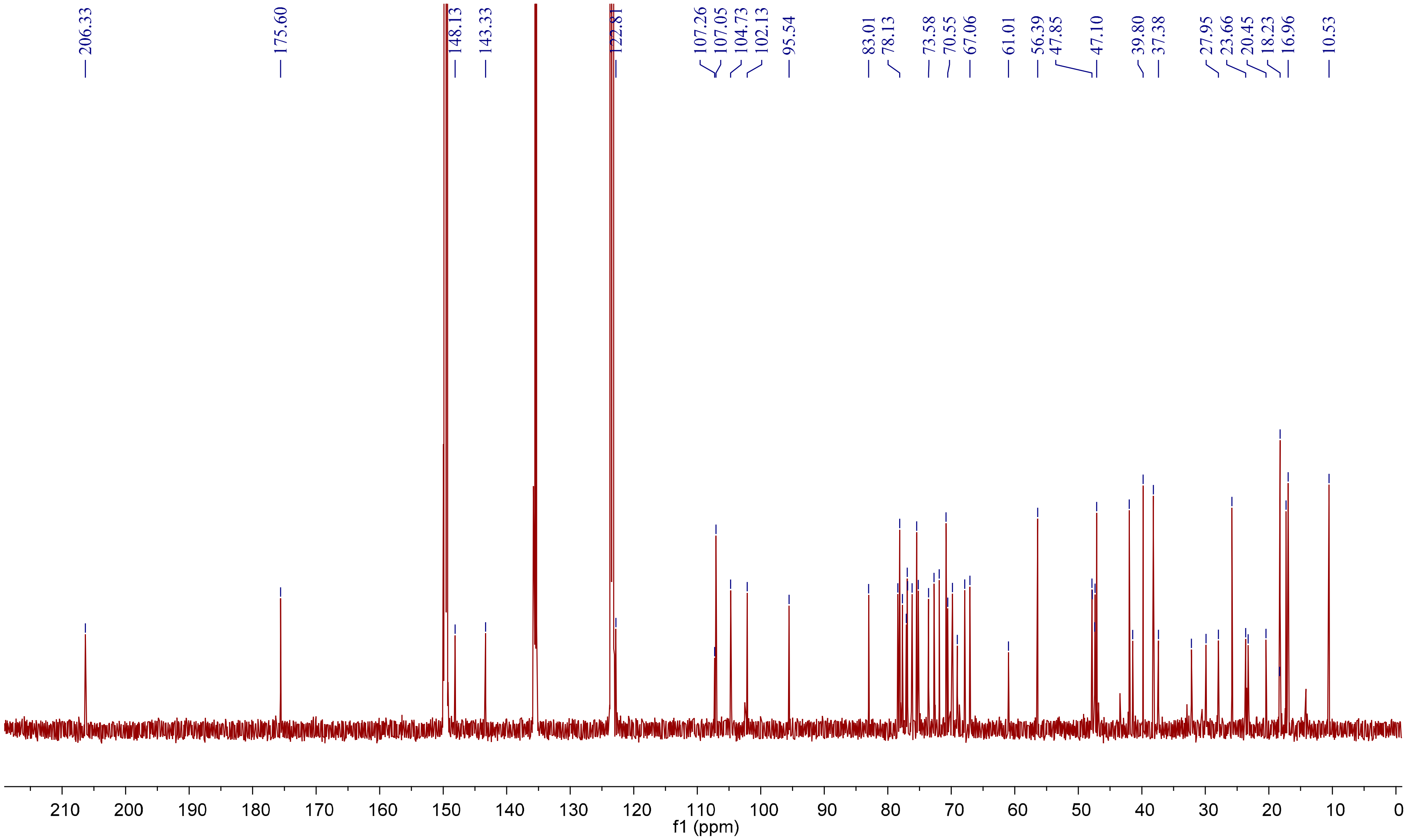

Figure S9. ^{13}C-NMR spectrum of compound **2**.

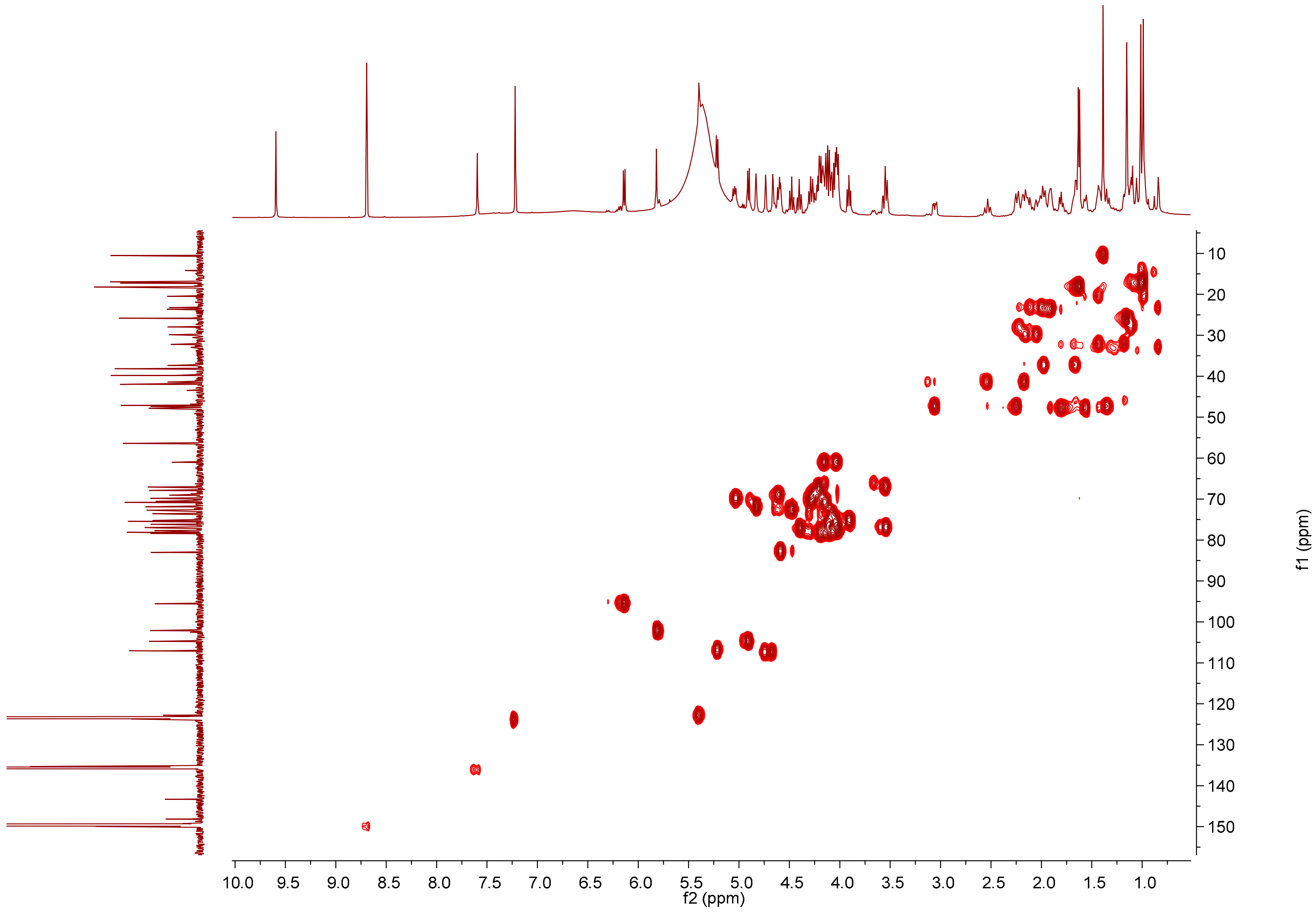

Figure S10. HSQC spectrum of compound **2**.

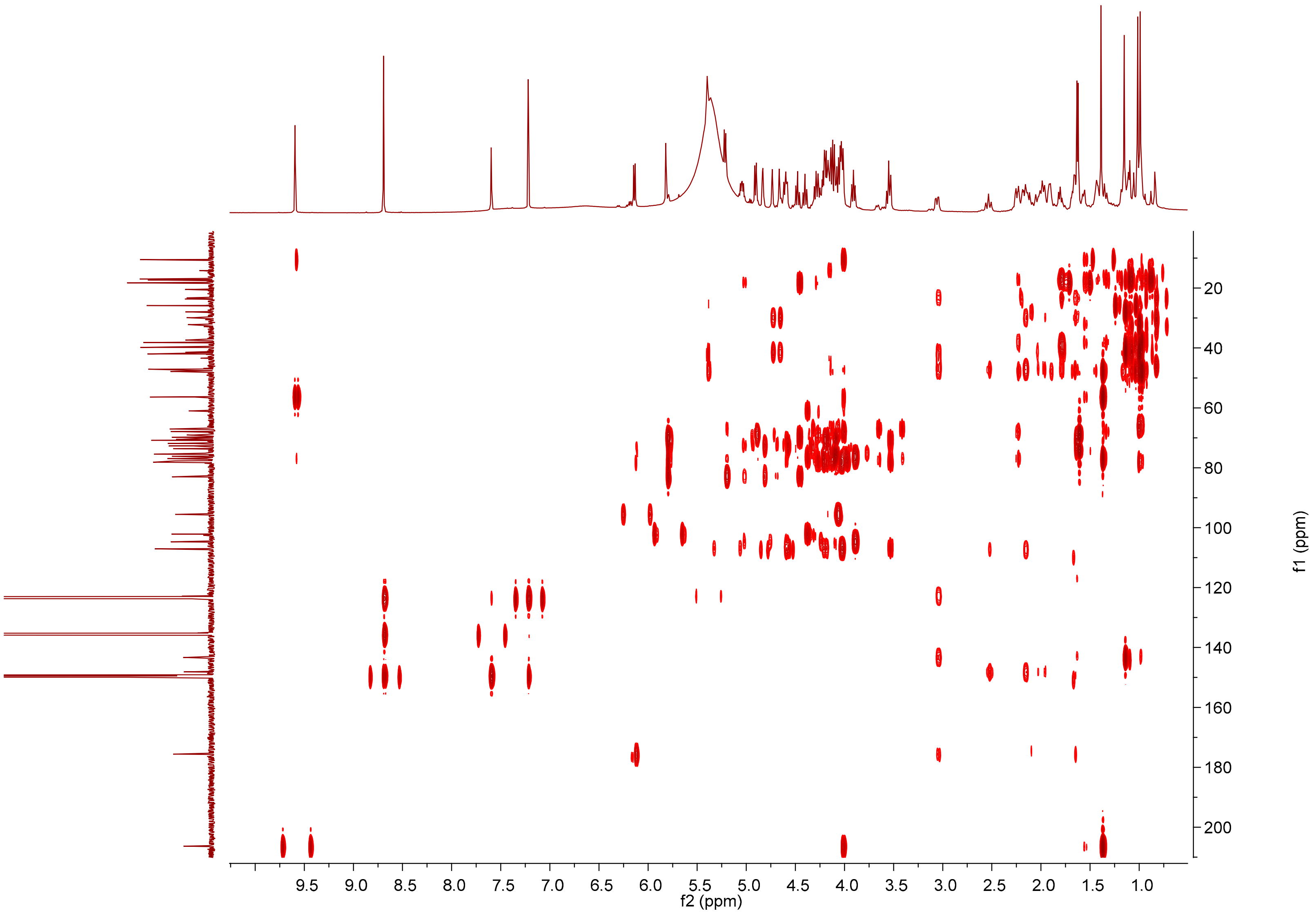

Figure S11. HMBC spectrum of compound **2**.

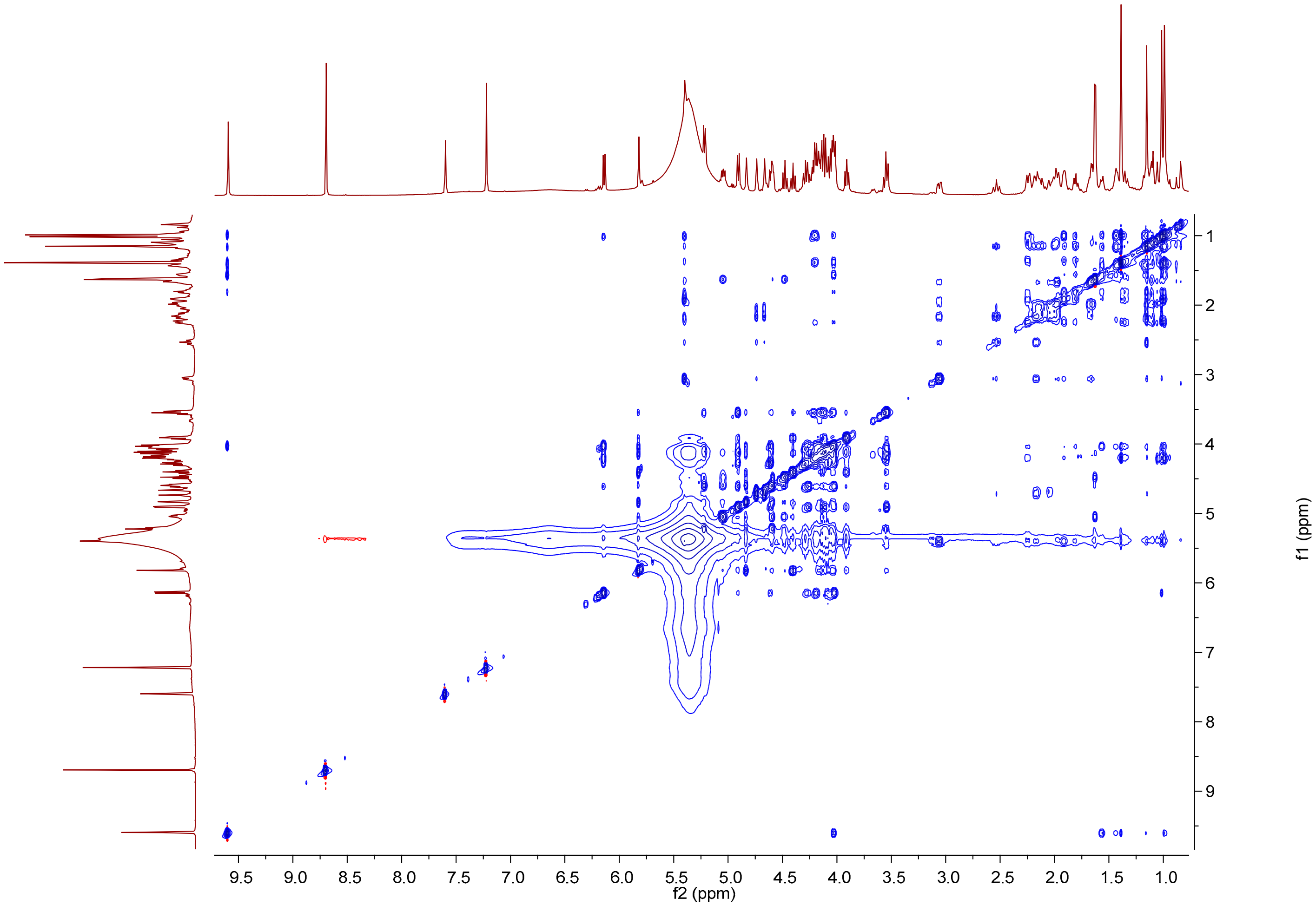

Figure S12. NOESY spectrum of compound **2**.
